# Radon Control Activities for Lung Cancer Prevention in National Comprehensive Cancer Control Program Plans, 2005–2011

**DOI:** 10.5888/pcd10.120337

**Published:** 2013-08-08

**Authors:** Antonio Neri, Sherri L. Stewart, William Angell

**Affiliations:** Author Affiliations: Sherri L. Stewart, Centers for Disease Control and Prevention, Atlanta, Georgia; William Angell, University of Minnesota, St. Paul, Minnesota.

## Abstract

**Introduction:**

Radon is the second leading cause of lung cancer among smokers and the leading cause among nonsmokers. The US Environmental Protection Agency recommends that every home be tested for radon. Comprehensive Cancer Control (CCC) programs develop cancer coalitions that coordinate funding and resources to focus on cancer activities that are recorded in cancer plans. Radon tests, remediation, and radon mitigation techniques are relatively inexpensive, but it is unclear whether coalitions recognize radon as an important carcinogen.

**Methods:**

We reviewed 65 cancer plans created from 2005 through 2011 for the terms “radon,” “radiation,” or “lung.” Plan activities were categorized as radon awareness, home testing, remediation, supporting radon policy activities, or policy evaluation. We also reviewed each CCC program’s most recent progress report. Cancer plan content was reviewed to assess alignment with existing radon-specific policies in each state.

**Results:**

Twenty-seven of the plans reviewed (42%) had radon-specific terminology. Improving awareness of radon was included in all 27 plans; also included were home testing (n = 21), remediation (n = 11), support radon policy activities (n = 13), and policy evaluation (n = 1). Three plans noted current engagement in radon activities. Thirty states had radon-specific laws; most (n = 21) were related to radon professional licensure. Eleven states had cancer plan activities that aligned with existing state radon laws.

**Conclusion:**

Although several states have radon-specific policies, approximately half of cancer coalitions may not be aware of radon as a public health issue. CCC-developed cancer coalitions and plans should prioritize tobacco control to address lung cancer but should consider addressing radon through partnership with existing radon control programs.

## Introduction

Lung cancer is the leading cause of cancer death in the United States, and smoking is the strongest risk factor for the disease (1). Exposure to radon is the second leading risk factor, causing approximately 21,000 cases of lung cancer per year (2–8). Analyses of pooled data from multiple studies of home-based radon health outcomes in China, Europe, and North America have found that people with lung cancer were more likely to be exposed to radon in their homes than those without lung cancer. Most of these studies concluded that radon is an independent risk factor for lung cancer, regardless of smoking status (9–11).

Radon is a colorless, odorless, radioactive gas that is a decay product of uranium. Radon is often found at higher concentrations in the lower levels of buildings than in upper levels. The US Surgeon General and the Environmental Protection Agency (EPA) estimate that 1 in 15 residences in the United States exceed the 4.0 pCi/L (picocuries per liter of air, the standard US metric for radon) EPA action level for radon and recommend that every residence be tested for radon (12,13). Several states have radon-related laws (14) (Table 1). EPA funds state and tribal radon control programs to subsidize or encourage radon testing in residences and schools, mitigate residences with high radon levels, encourage radon-resistant building practices, and develop professional licensure programs (www.epa.gov/radon/sirgprogram.html). The National Radon Proficiency Program is the voluntary national certification program for radon professionals; it is currently administered through the American Association of Radon Scientists and Technologists (http://nrpp.info/).

**Table 1:** Radon-Related Policies by State as of 2012^a^

State	Radon Policies in Effect
Alabama	None
Alaska	None
Arizona	None
Arkansas	None
California	Radon professional licensing, radon building code for new homes
Colorado	Radon testing in schools
Connecticut	Radon professional licensing, radon building code for new schools, radon testing in day care centers, radon testing in schools
Delaware	Radon-specific notification during home sales
District of Columbia	Radon professional licensing
Florida	Radon professional licensing, radon building code for new homes, radon testing in schools, radon testing in day care centers, radon testing in government-owned buildings, radon-specific notification during home sales, radon-specific notification in home leasing
Georgia	None
Hawaii	None
Idaho	Radon testing in day care centers
Illinois	Radon professional licensing, radon building code for new schools, radon testing in schools, radon-specific notification during home sales, radon-specific notification in home leasing
Indiana	Radon professional licensing
Iowa	Radon professional licensing, radon testing in schools; radon testing in day care centers, radon-specific notification during home sales
Kansas	Radon professional licensing, radon-specific notification during home sales
Kentucky	Radon professional licensing
Louisiana	None
Maine	Radon professional licensing, radon building code for new homes, radon-specific notification in home leasing
Maryland	Radon professional licensing, radon testing in day care centers
Massachusetts	Radon-specific notification during home sales
Michigan	Radon building code for new homes, radon testing in day care centers
Minnesota	Radon building code for new homes, radon building code for new government-owned buildings
Mississippi	None
Missouri	None
Montana	Radon professional licensing, radon-specific notification during home sales
Nebraska	Radon professional licensing
Nevada	Radon professional licensing, radon-specific notification during home sales
New Hampshire	Radon testing in schools, radon testing in government-owned buildings, radon-specific notification during home sales
New Jersey	Radon professional licensing, radon building code for new homes, radon building code for new schools, radon testing in day care centers, radon-specific notification during home sales, radon-specific notification in home leasing.
New Mexico	None
New York	Radon professional licensing, radon testing in schools
North Carolina	None
North Dakota	None
Ohio	Radon professional licensing
Oklahoma	None
Oregon	Radon building code for new homes
Pennsylvania	Radon professional licensing
Rhode Island	Radon professional licensing, radon building code for new schools, radon testing in day care centers, radon testing in schools, radon testing in government-owned buildings, radon-specific notification during home sales
South Carolina	None
South Dakota	None
Tennessee	None
Texas	None
Utah	Radon professional licensing
Virginia	Radon professional licensing, radon building code for new homes, radon building code for new government-owned buildings, radon testing in schools
Vermont	None
Washington	Radon building code for new homes
West Virginia	Radon professional licensing, radon building code for new schools, radon testing in schools
Wisconsin	None
Wyoming	None

a 30 states and Washington, DC, had some type of radon law in place as of February 2012 (14).

The National Comprehensive Cancer Control Program (NCCCP) is administered by the Centers for Disease Control and Prevention (CDC) and funds Comprehensive Cancer Control (CCC) programs in 65 states, tribes, and territories to form cancer coalitions (15). These coalitions, supported through a variety of federal and nonfederal sources and volunteer activity, work to synergize efforts to prevent and control cancer in their populations (15). The objective of this study was to review current cancer plans and policies to determine whether states, tribes, and territories were aware of radon as a health issue and what radon-specific activities were planned or completed to prevent radon-related lung cancer.

## Methods

Cancer plans for all NCCCP-funded programs are publicly available on the Cancer Control P.L.A.N.E.T website (http://cancercontrolplanet.cancer.gov), and the search tool on this website was used to search plans. The search was conducted in January 2011, at which time 57 of the 65 NCCCP-funded programs had plans that spanned 2005 through 2010, and 8 programs had plans that spanned 2007 through 2012.

The terms “radon,” “radiation,” or “lung,” were each used independently to search plans. Each plan identified as having those terms was then reviewed by using the Adobe Acrobat Reader version 9.4.0 (Adobe Systems Inc, San Jose, California) search tool for the same terms within the document. All sections pertaining to lung cancer or environmental health were reviewed for any possible connection to radon testing or mitigation. Pertinent sections of each cancer plan were copied into a Microsoft Access (Microsoft Corporation, Redmond, Washington) database. In review, each section was classified as pertaining to 1 or more of the following categories: improving awareness of radon as an important carcinogen, activities to test residences for radon, activities to mitigate existing residences with high radon levels, supporting radon policy activities, and efforts to support radon policy activities. Frequencies of each activity were calculated using the query function in Microsoft Access. One investigator (A.N.) conducted all searches and categorization of terms.

Interim progress reports from CCC programs for one 6-month period of the CDC grant cycle (June 2010 through December 2010) were also reviewed to determine whether radon-specific activities identified in plans had been or were being implemented at that time. Interim progress reports from programs with plans that identified an action pertaining to radon were reviewed for the same terms to determine which activities had been undertaken.

Finally, the 2012 Environmental Law Institute compilation of all state radon laws were reviewed to classify each state’s radon law into the following categories: state-based licensing of radon professionals; radon building codes for new residences or schools; radon testing in residences, schools, day-care facilities, and government-owned buildings; signed notification of radon testing in residence sales or leases, and general radon education. A comparison of how a state’s cancer plan activities aligned with current radon laws was made.

## Results

All 65 cancer plans reviewed contained the word “lung,” 59 plans contained the word “radiation,” and 33 plans contained the word “radon.” Investigators identified 42 plans (65% of all plans) that had terminology potentially associated with radon. Further review found that 15 plans either identified radon as a cancer-causing agent but did not discuss radon-related actions (7 plans) or had actions that were too ambiguous to classify (8 plans).

**Figure 1:**
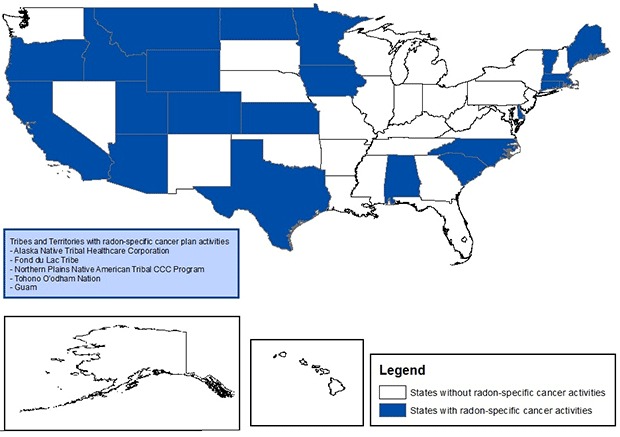
States with radon-specific activities in cancer plans. The tribes and territories with radon-specific activities are Alaska Native Tribal Healthcare Corporation, Fond du Lac Tribe, Guam, Northern Plains Native American Tribal Comprehensive Cancer Control Program, and Tohono O’odham Nation.

Twenty-seven plans (42%) were found to have measurable activities specific to radon (Figure 1). All 27 had activities in place to improve awareness of radon as a risk factor for lung cancer. Most of these plan activities were for education campaigns; only 3 plans mentioned partnership with state and non-government programs for increasing awareness. Twenty-one plans (32%) had activities to increase residential radon testing (Table 2). Most of these plan activities had general statements about testing, and 4 plans mentioned partnership with state and nongovernment agencies involved with radon. Eleven plans sought to promote remediation of homes with high radon levels, and 7 of these mentioned partnership with other programs engaged in radon activities. Thirteen plans supported radon policy activities, 8 of which mentioned partnership with other agencies working to address radon. Finally, 1 plan included activities to evaluate radon policies.

**Table 2:** Radon-Related Activities for Lung Cancer Prevention in National Comprehensive Cancer Control Program Plans (N = 27), 2005–2011

Category	n (%)	Representative Quotes From Plans in the Category
Improve awareness of radon	25 (38%)	“Inform people who live in radon-prone areas about the risks of radon and ways to reduce those risks”
Improve home testing	21 (32%)	“Identify homes with high radon levels through home testing.”
Promote remediation of high radon homes	11 (17%)	“[Program] . . . has trained several contractors in radon home remediation.”
Support radon policy activities^a^	13 (20%)	“Support the [Program’s] Environmental Protection Agency in their efforts to . . . require radon testing for all homes, public buildings, and commercial buildings prior to being built or sold for real estate transactions.”
Evaluate existing radon policy	1 (2%)	“Ensure the compatibility of state and federal information and regulations on carcinogens in the workplace, with continuing reviews of policies of all levels of government.”

a To the best of the authors’ knowledge, no federal funds were used to advocate for legislative or policy changes.

Of the 27 cancer plans that mentioned radon, 3 (11%) noted ongoing radon-specific activities in their action plans or 2010 interim progress reports submitted to CDC. These activities were related to education, policy evaluation, and test-kit distribution. The 2010 interim progress reports for the remaining 24 CCC programs whose cancer plans mentioned radon gave no indication of completed or planned radon activities, indicating that such activities had not been undertaken in the last year.

**Figure 2:**
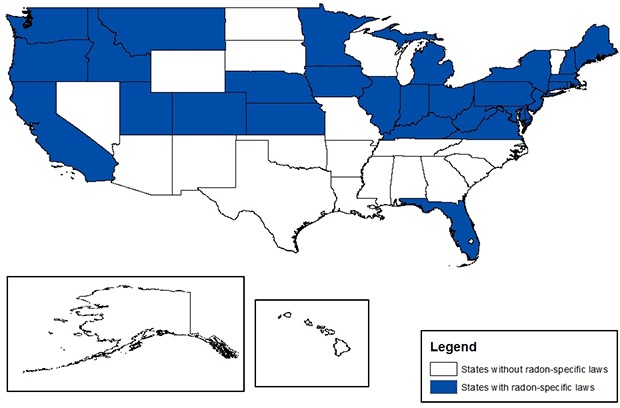
States with radon-specific laws.

As of February 2012, 30 states and Washington, DC, had radon-specific laws (14) (Figure 2). Most (n = 21) were related to state-based licensing of radon professionals. Other laws included radon testing in residences, schools, day care facilities, and government-owned buildings (n = 12); radon building codes for new residences or schools (n=14); and signed notification of radon testing in residential sales or leases (n = 12).

Of the 27 states with cancer plan activities specific to radon, 11 (41%) were aligned with a radon-specific law related to raising awareness of radon as an issue, 4 (15%) had both a cancer plan activity and a law related to increasing testing, and 9 (33%) had aligned activities and laws related to radon mitigation.

## Discussion

Of the 65 NCCCP plans reviewed, only 42% (n = 27) mentioned radon-related activities for prevention of lung cancer. Radon is a well-established cause of lung cancer that is relatively easy and inexpensive to test for, mitigate, or prevent exposure to through low-cost, radon-resistant building practices (12,13). Local soil composition, foundation type and air sealing, indoor–soil air-pressure differences, and many other characteristics determine radon concentrations within homes. Efforts to enhance residential energy efficiency need to be approached with radon in mind as these may alter home indoor–outdoor-soil air exchange rates and thus affect the indoor concentration of radon and other air pollutants (16–19). In addition to the EPA radon program, costs for testing or mitigation may be subsidized by the US Department of Housing and Urban Development’s 203(k) mortgage financing program (20) and the US Department of Energy’s Weatherization and Intergovernmental Program (http://www1.eere.energy.gov/wip). State-based cancer and radon programs may also find useful agency activities in the EPA-led Federal Radon Action Plan. This plan coordinates radon efforts of both federal and nonfederal agencies; the plan and state-specific radon contacts are available through the EPA radon program website (http://www.epa.gov/radon/).

CCC programs are ideally positioned to leverage existing resources to help educate residents, test all residences and buildings (particularly schools), remediate residences where necessary, and educate leaders about radon testing and radon-resistant construction designs in all buildings. Also, efforts made toward radon testing and mitigation are readily measureable and easily evaluated. All 3 cancer plans that noted current actions were able to measure the effect of efforts through educational sessions, policy evaluation, or radon test-kit distribution. Radon mitigation activities would therefore easily address 2 priority areas for NCCCP-funded programs: emphasizing the primary prevention of cancer, and demonstrating outcomes through evaluation (21).

Tobacco use and second-hand smoke exposure account for most lung cancers. Cancer control programs should continue to prioritize tobacco-use prevention and cessation efforts to reduce lung cancer incidence, as noted in a recent comparison of radon versus smoking (6). However, a role remains for addressing radon in addition to tobacco-use prevention and cessation efforts.

Radon interventions can be cost-effective. Although there is no US consensus on thresholds for cost-effectiveness of an intervention, the World Health Organization recommendation is that an intervention be considered highly cost-effective if it costs less than the country’s per-capita gross domestic product (GDP), cost effective if it costs between 1 and 3 times the country’s per-capita GDP, and not cost effective if it costs more than 3 times the country’s per-capita GDP (22). If one were to use these thresholds with the 2008 US per-capita GDP ($38,262) the cutoffs would be less than $38,262, $38,262 to $114,786, and more than $114,786, respectively (23). Estimates for radon universal identification and mitigation costs in various studies ranged from US $25,698 to $715,193 per life-year gained and from US $36,837 to $76,444 per quality-adjusted life-year gained (24–26). All reported costs here are for mitigation of existing homes, a conservative estimate when compared with lower costs for new homes. Additionally, costs are converted to US currency if necessary by using the average conversion rate of the year of study and then converted to 2008 US dollars using the Consumer Price Index (27). In more practical terms, these findings make sense: a 1-time home radon test kit costs about $15 per home or less (2012 dollars), remediation costs vary from $300 to $2,500 (if needed at all as the EPA estimates that only 1 in 15 residences has elevated radon levels), and radon mitigation systems in new residences that prevent exposure have been shown to cost between $250 to $3,000 per structure (12). This investment in radon mitigation systems benefits current residents as well as future occupants of the house.

This study has several limitations. NCCCP-funded programs and their coalitions regularly update their cancer plans and this review only looked at a short time period. Cancer plans are an indicator of areas of interest and focus, but they do not always represent activities that have actually been undertaken. Although we were able to review 1 series of interim progress reports for a 5-year time period, activities accomplished prior to this time period may not be included in these interim progress reports for 1 year. However, most NCCCP-funded programs continue to report accomplishments within the last 5 years, and these would likely be identified by reviewing the most recent program progress report. In addition, only 1 author determined whether programs were addressing radon and categorized program activities. It is unlikely, however, that adding a second independent reviewer would have dramatically changed the results, and the methods used here are consistent with those of other NCCCP content analyses (28,29).

Radon is a well-known lung carcinogen. EPA guidelines for action exist, a low-cost radon test is available, and remediation and prevention is relatively inexpensive. However, 58% of cancer plans implemented between 2005 and 2011 did not incorporate radon-specific activities, even though EPA-funded radon control programs exist in most states. State-level radon programs and NCCCP-funded programs should consider collaborative efforts to leverage existing resources in identifying and controlling radon exposure. In addition, cancer programs should consider radon activities to prevent lung cancer as they routinely update their cancer plans. Research to identify which radon interventions and methods of implementation have the greatest effect in particular populations would greatly benefit programs working to address radon efficiently and comprehensively.
